# Ethyl 4-amino-3-methyl­benzoate

**DOI:** 10.1107/S1600536808005989

**Published:** 2008-04-02

**Authors:** Wen-Lan Song, Dan Wang, Xin-Hua Li, De-Cai Wang

**Affiliations:** a Department of Pharmaceutical Engineering, College of Life Sciences and Pharmaceutical Engineering, Nanjing University of Technolgy, Xinmofan Road No. 5, Nanjing 210009, People’s Republic of China; b Bioengineering Department, Xuzhou Higher Vocational College of Bioengineering, Mine West Road, Xuzhou, Xuzhou 221006, People’s Republic of China

## Abstract

The asymmetric unit of the title compound, C_10_H_13_NO_2_, contains two mol­ecules which are linked *via* an N—H⋯N hydrogen bonds to form a dimer. These dimers are further linked *via* N—H⋯O inter­molecular hydrogen bonds.

## Related literature

For related literature, see: Baraldi *et al.* (1999 ▶, 2000 ▶, 2003 ▶, 2007 ▶); Wang *et al.* (2003 ▶); Zaffaroni *et al.* (2002 ▶). For bond-length data, see: Allen *et al.* (1987 ▶).
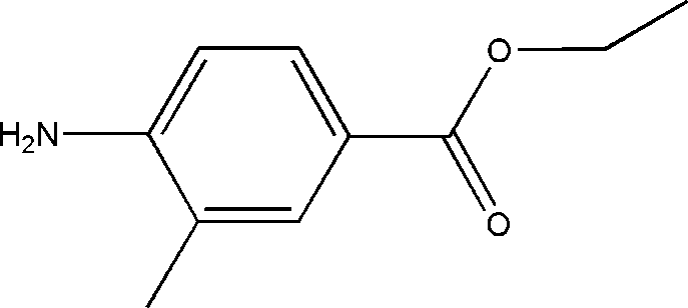


## Experimental

### 

#### Crystal data

C_10_H_13_NO_2_
*M* *_r_* = 179.21
Triclinic, 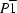

*a* = 8.0110 (16) Å
*b* = 8.7030 (17) Å
*c* = 15.835 (3) Å
α = 90.78 (3)°
β = 95.13 (3)°
γ = 114.34 (3)°
*V* = 1000.3 (3) Å^3^
*Z* = 4
Mo *K*α radiation
μ = 0.08 mm^−1^
*T* = 298 (2) K
0.30 × 0.20 × 0.10 mm

#### Data collection

Enraf–Nonius CAD-4 diffractometer
Absorption correction: ψ scan (North *et al.*, 1968 ▶) *T*_min_ = 0.975, *T*_max_ = 0.991
3874 measured reflections
3594 independent reflections
2146 reflections with *I* > 2σ(*I*)
*R*_int_ = 0.052
3 standard reflections every 200 reflections intensity decay: none

#### Refinement

*R*[*F* ^2^ > 2σ(*F* ^2^)] = 0.074
*wR*(*F* ^2^) = 0.176
*S* = 1.02
3594 reflections
235 parameters
H-atom parameters constrained
Δρ_max_ = 0.23 e Å^−3^
Δρ_min_ = −0.23 e Å^−3^

### 

Data collection: *CAD-4 Software* (Enraf–Nonius, 1989 ▶); cell refinement: *CAD-4 Software*; data reduction: *XCAD4* (Harms & Wocadlo,1995 ▶); program(s) used to solve structure: *SHELXS97* (Sheldrick, 2008 ▶); program(s) used to refine structure: *SHELXL97* (Sheldrick, 2008 ▶); molecular graphics: *SHELXTL* (Sheldrick, 2008 ▶); software used to prepare material for publication: *SHELXL97*

## Supplementary Material

Crystal structure: contains datablocks I, global. DOI: 10.1107/S1600536808005989/er2050sup1.cif

Structure factors: contains datablocks I. DOI: 10.1107/S1600536808005989/er2050Isup2.hkl

Additional supplementary materials:  crystallographic information; 3D view; checkCIF report

## Figures and Tables

**Table 1:** Hydrogen-bond geometry (Å, °)

*D*—H⋯*A*	*D*—H	H⋯*A*	*D*⋯*A*	*D*—H⋯*A*
N1—H1*A*⋯O2^i^	0.86	2.33	3.023 (5)	138
N1—H1*B*⋯N2	0.86	2.61	3.242 (5)	131
N2—H2*C*⋯O2^i^	0.86	2.35	3.160 (4)	157
N2—H2*D*⋯O4^ii^	0.86	2.15	2.967 (4)	158

Symmetry codes: (i) 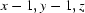; (ii) 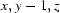.

**Fig. 1.**
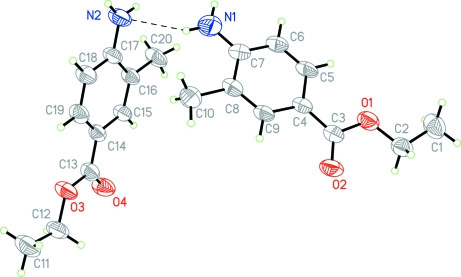
A view of the molecular structure of (I), showing displacement ellipsoids at the 50% probability level. N—H···N hydrogen bonds are shown as dashed lines.

## supplementary crystallographic information

### Comment

Ethyl 3-methyl-4-aminobenzoate is a material for preparing the important intermidates of bis-(2-haloethyl)aminophenyl substituted distamycin derivatives(Baraldi *et al.*, 2007), which are used as antitumor alkylating and antiviral agents related to the known antibiotic distamycin A (Baraldi *et al.*, 1999). Distamycin A belongs to the family of the pyrroleamidine antibiotics (Baraldi *et al.*, 2000) and is reported to interact reversibly and selectively with DNA-AT sequences interfering with both replication and transcription (Baraldi *et al.*, 2003). Bis-(2-haloethyl)aminophenyl substituted distamycin derivatives can therefore be used in a treatment to ameliorate a cancer (Wang *et al.*, 2003). They may be administered to improve the condition of a patient having a leukaemia lymphoma, sarcoma, such as myeloblastic leukaemia, neuroblastoma, Wilm's tumor or malignant neoplasm of the bladder, breast, lung or thyroid. (Zaffaroni *et al.*, 2002).

The molecular structure of (I) is shown in Fig. 1. The bond lengths and angles are within normal ranges (Allen *et al.*, 1987).

The asymmetric unit of the title compound, contains two molecules which are linked *via* N—H···N hydrogen bonds to form dimers. In the crystals, molecules are linked *via* N—H···O Intermolecular hydrogen bonds (Table 1), which may be effective in the stabilization of the crystals.

### Experimental

The title compound was prepared from 3-Methyl-4-aminobenzoic acid (15.2 g, 100 mmole) in ethanol (40.4 ml, 1000 mmole). After the solid has melted, concentrated hydrochloric acid (142 g, 120 ml) was added dropwise from a dropping funnel at 90°C, the the reaction mixture was cooled with ice and water and finaly the product was filtered by suction. Suitable crystals were obtained by evaporation of a methanol solution for about 3 d.

### Refinement

All H atoms were placed geometrically at the distances of 0.93–0.97 Å for C—H and 0.86 Å for N—H and included in the refinement in riding motion approximation with *U*_iso_(H) = 1.2 or 1.5*U*_eq_ of the carrier atom.

### Crystal data

**Table:**

C_10_H_13_NO_2_	*Z* = 4
*M**_r_* = 179.21	*F*_000_ = 384
Triclinic, *P*1	*D*_x_ = 1.190 Mg m^−^^3^
Hall symbol: -P 1	Mo *K*α radiation λ = 0.71073 Å
*a* = 8.0110 (16) Å	Cell parameters from 25 reflections
*b* = 8.7030 (17) Å	θ = 10–14º
*c* = 15.835 (3) Å	µ = 0.08 mm^−^^1^
α = 90.78 (3)º	*T* = 298 (2) K
β = 95.13 (3)º	Block, colorless
γ = 114.34 (3)º	0.30 × 0.20 × 0.10 mm
*V* = 1000.3 (3) Å^3^	

### Data collection

**Table:**

Enraf–Nonius CAD-4 diffractometer	*R*_int_ = 0.052
Radiation source: fine-focus sealed tube	θ_max_ = 25.2º
Monochromator: graphite	θ_min_ = 1.3º
*T* = 298(2) K	*h* = −9→9
ω/2θ scans	*k* = −10→10
Absorption correction: ψ scan(North *et al.*, 1968)	*l* = 0→18
*T*_min_ = 0.975, *T*_max_ = 0.991	3 standard reflections
3874 measured reflections	every 200 reflections
3594 independent reflections	intensity decay: none
2146 reflections with *I* > 2σ(*I*)	

### Refinement

**Table:**

Refinement on *F*^2^	Secondary atom site location: difference Fourier map
Least-squares matrix: full	Hydrogen site location: inferred from neighbouring sites
*R*[*F*^2^ > 2σ(*F*^2^)] = 0.075	H-atom parameters constrained
*wR*(*F*^2^) = 0.177	*w* = 1/[σ^2^(*F*_o_^2^) + (0.04*P*)^2^ + 1.2*P*] where *P* = (*F*_o_^2^ + 2*F*_c_^2^)/3
*S* = 1.02	(Δ/σ)_max_ < 0.001
3594 reflections	Δρ_max_ = 0.23 e Å^−^^3^
235 parameters	Δρ_min_ = −0.22 e Å^−^^3^
Primary atom site location: structure-invariant direct methods	Extinction correction: none

### Special details

**Table:**

Geometry. All e.s.d.'s (except the e.s.d. in the dihedral angle between two l.s. planes) are estimated using the full covariance matrix. The cell e.s.d.'s are taken into account individually in the estimation of e.s.d.'s in distances, angles and torsion angles; correlations between e.s.d.'s in cell parameters are only used when they are defined by crystal symmetry. An approximate (isotropic) treatment of cell e.s.d.'s is used for estimating e.s.d.'s involving l.s. planes.
Refinement. Refinement of *F*^2^ against ALL reflections. The weighted *R*-factor *wR* and goodness of fit *S* are based on *F*^2^, conventional *R*-factors *R* are based on *F*, with *F* set to zero for negative *F*^2^. The threshold expression of *F*^2^ > σ(*F*^2^) is used only for calculating *R*-factors(gt) *etc*. and is not relevant to the choice of reflections for refinement. *R*-factors based on *F*^2^ are statistically about twice as large as those based on *F*, and *R*- factors based on ALL data will be even larger.

### Fractional atomic coordinates and isotropic or equivalent isotropic displacement parameters (Å^2^)

**Table:**

	*x*	*y*	*z*	*U*_iso_*/*U*_eq_	
O1	1.2476 (4)	0.4839 (3)	1.01662 (15)	0.0684 (7)	
O2	1.2577 (4)	0.5912 (3)	0.88836 (15)	0.0742 (8)	
N1	0.5942 (5)	−0.1423 (4)	0.8227 (2)	0.0872 (11)	
H1A	0.5484	−0.2192	0.8580	0.105*	
H1B	0.5509	−0.1604	0.7701	0.105*	
C1	1.4390 (7)	0.6205 (6)	1.1432 (2)	0.0884 (13)	
H1C	1.5389	0.7220	1.1674	0.133*	
H1D	1.4718	0.5267	1.1505	0.133*	
H1E	1.3311	0.6012	1.1712	0.133*	
C2	1.3999 (6)	0.6384 (5)	1.0501 (2)	0.0695 (10)	
H2A	1.3681	0.7338	1.0420	0.083*	
H2B	1.5077	0.6571	1.0211	0.083*	
C3	1.1842 (5)	0.4730 (4)	0.9338 (2)	0.0549 (8)	
C4	1.0343 (5)	0.3145 (4)	0.9073 (2)	0.0514 (8)	
C5	0.9506 (5)	0.1886 (4)	0.9638 (2)	0.0620 (10)	
H5A	0.9932	0.2072	1.0212	0.074*	
C6	0.8093 (5)	0.0413 (4)	0.9358 (2)	0.0629 (10)	
H6A	0.7590	−0.0410	0.9743	0.075*	
C7	0.7362 (5)	0.0087 (4)	0.8503 (2)	0.0607 (9)	
C8	0.8176 (5)	0.1308 (4)	0.7910 (2)	0.0579 (9)	
C9	0.9597 (5)	0.2792 (4)	0.8216 (2)	0.0537 (8)	
H9A	1.0103	0.3620	0.7834	0.064*	
C10	0.7439 (6)	0.0996 (5)	0.6984 (2)	0.0790 (12)	
H10A	0.8184	0.1926	0.6672	0.119*	
H10B	0.6192	0.0889	0.6924	0.119*	
H10C	0.7471	−0.0026	0.6767	0.119*	
O3	0.2589 (4)	0.2954 (3)	0.47872 (15)	0.0695 (7)	
O4	0.1772 (4)	0.3743 (3)	0.59841 (17)	0.0836 (9)	
N2	0.2153 (5)	−0.3189 (4)	0.69728 (19)	0.0828 (11)	
H2C	0.1979	−0.3380	0.7496	0.099*	
H2D	0.2337	−0.3903	0.6656	0.099*	
C11	0.3055 (7)	0.4392 (6)	0.3509 (3)	0.0990 (15)	
H11A	0.3072	0.5370	0.3229	0.149*	
H11B	0.2152	0.3391	0.3204	0.149*	
H11C	0.4248	0.4375	0.3523	0.149*	
C12	0.2570 (6)	0.4456 (5)	0.4406 (2)	0.0764 (11)	
H12A	0.3465	0.5466	0.4722	0.092*	
H12B	0.1360	0.4461	0.4403	0.092*	
C13	0.2119 (5)	0.2726 (5)	0.5596 (2)	0.0608 (9)	
C14	0.2194 (5)	0.1199 (4)	0.59291 (19)	0.0543 (8)	
C15	0.1854 (5)	0.0871 (4)	0.6782 (2)	0.0545 (8)	
H15A	0.1632	0.1649	0.7108	0.065*	
C16	0.1840 (5)	−0.0559 (4)	0.7146 (2)	0.0549 (8)	
C17	0.2137 (5)	−0.1753 (4)	0.6644 (2)	0.0597 (9)	
C18	0.2476 (6)	−0.1425 (5)	0.5793 (2)	0.0682 (11)	
H18A	0.2686	−0.2200	0.5458	0.082*	
C19	0.2499 (5)	0.0029 (5)	0.5451 (2)	0.0660 (10)	
H19A	0.2724	0.0221	0.4887	0.079*	
C20	0.1455 (6)	−0.0876 (5)	0.8067 (2)	0.0768 (12)	
H20A	0.1279	0.0055	0.8312	0.115*	
H20B	0.2479	−0.0986	0.8379	0.115*	
H20C	0.0363	−0.1897	0.8089	0.115*	

### Atomic displacement parameters (Å^2^)

**Table:**

	*U*^11^	*U*^22^	*U*^33^	*U*^12^	*U*^13^	*U*^23^
O1	0.0901 (18)	0.0660 (16)	0.0515 (14)	0.0347 (14)	0.0056 (13)	0.0157 (12)
O2	0.100 (2)	0.0594 (15)	0.0596 (16)	0.0282 (14)	0.0118 (14)	0.0263 (13)
N1	0.099 (3)	0.072 (2)	0.078 (2)	0.021 (2)	0.013 (2)	0.0202 (18)
C1	0.114 (4)	0.099 (3)	0.063 (3)	0.059 (3)	−0.010 (2)	−0.008 (2)
C2	0.090 (3)	0.060 (2)	0.067 (2)	0.040 (2)	0.000 (2)	0.0075 (19)
C3	0.068 (2)	0.055 (2)	0.051 (2)	0.0334 (18)	0.0094 (17)	0.0118 (16)
C4	0.068 (2)	0.0485 (19)	0.0467 (18)	0.0319 (17)	0.0108 (16)	0.0142 (15)
C5	0.087 (3)	0.060 (2)	0.048 (2)	0.037 (2)	0.0177 (18)	0.0259 (17)
C6	0.078 (3)	0.057 (2)	0.057 (2)	0.030 (2)	0.0191 (19)	0.0255 (18)
C7	0.072 (2)	0.049 (2)	0.067 (2)	0.0285 (18)	0.0145 (19)	0.0180 (17)
C8	0.073 (2)	0.062 (2)	0.049 (2)	0.0373 (19)	0.0100 (17)	0.0149 (17)
C9	0.073 (2)	0.0505 (19)	0.0439 (18)	0.0305 (18)	0.0133 (16)	0.0173 (15)
C10	0.096 (3)	0.076 (3)	0.056 (2)	0.026 (2)	0.006 (2)	0.012 (2)
O3	0.0994 (19)	0.0705 (16)	0.0524 (15)	0.0462 (15)	0.0200 (13)	0.0246 (12)
O4	0.140 (3)	0.0684 (17)	0.0626 (17)	0.0603 (18)	0.0224 (16)	0.0110 (14)
N2	0.148 (3)	0.070 (2)	0.0511 (18)	0.065 (2)	0.0102 (19)	0.0136 (16)
C11	0.135 (4)	0.104 (4)	0.066 (3)	0.053 (3)	0.022 (3)	0.038 (3)
C12	0.102 (3)	0.068 (2)	0.066 (3)	0.041 (2)	0.012 (2)	0.027 (2)
C13	0.077 (2)	0.064 (2)	0.047 (2)	0.034 (2)	0.0102 (17)	0.0113 (17)
C14	0.072 (2)	0.055 (2)	0.0380 (17)	0.0288 (17)	0.0062 (15)	0.0088 (15)
C15	0.079 (2)	0.056 (2)	0.0427 (18)	0.0406 (18)	0.0107 (16)	0.0082 (15)
C16	0.076 (2)	0.061 (2)	0.0390 (17)	0.0380 (18)	0.0113 (16)	0.0099 (15)
C17	0.094 (3)	0.055 (2)	0.0438 (19)	0.045 (2)	0.0064 (17)	0.0099 (15)
C18	0.117 (3)	0.064 (2)	0.0439 (19)	0.057 (2)	0.011 (2)	0.0021 (17)
C19	0.099 (3)	0.069 (2)	0.0382 (18)	0.041 (2)	0.0134 (18)	0.0084 (17)
C20	0.120 (3)	0.095 (3)	0.042 (2)	0.068 (3)	0.022 (2)	0.0199 (19)

### Geometric parameters (Å, °)

**Table:**

O1—C3	1.351 (4)	O3—C13	1.362 (4)
O1—C2	1.446 (4)	O3—C12	1.452 (4)
O2—C3	1.232 (4)	O4—C13	1.207 (4)
N1—C7	1.369 (5)	N2—C17	1.365 (4)
N1—H1A	0.8600	N2—H2C	0.8600
N1—H1B	0.8600	N2—H2D	0.8600
C1—C2	1.503 (5)	C11—C12	1.513 (5)
C1—H1C	0.9600	C11—H11A	0.9600
C1—H1D	0.9600	C11—H11B	0.9600
C1—H1E	0.9600	C11—H11C	0.9600
C2—H2A	0.9700	C12—H12A	0.9700
C2—H2B	0.9700	C12—H12B	0.9700
C3—C4	1.432 (5)	C13—C14	1.458 (5)
C4—C5	1.407 (4)	C14—C19	1.374 (5)
C4—C9	1.409 (4)	C14—C15	1.411 (4)
C5—C6	1.349 (5)	C15—C16	1.375 (4)
C5—H5A	0.9300	C15—H15A	0.9300
C6—C7	1.404 (5)	C16—C17	1.409 (4)
C6—H6A	0.9300	C16—C20	1.522 (4)
C7—C8	1.414 (5)	C17—C18	1.409 (4)
C8—C9	1.367 (5)	C18—C19	1.376 (5)
C8—C10	1.509 (5)	C18—H18A	0.9300
C9—H9A	0.9300	C19—H19A	0.9300
C10—H10A	0.9600	C20—H20A	0.9600
C10—H10B	0.9600	C20—H20B	0.9600
C10—H10C	0.9600	C20—H20C	0.9600
			
C3—O1—C2	118.2 (3)	C13—O3—C12	116.0 (3)
C7—N1—H1A	120.0	C17—N2—H2C	120.0
C7—N1—H1B	120.0	C17—N2—H2D	120.0
H1A—N1—H1B	120.0	H2C—N2—H2D	120.0
C2—C1—H1C	109.5	C12—C11—H11A	109.5
C2—C1—H1D	109.5	C12—C11—H11B	109.5
H1C—C1—H1D	109.5	H11A—C11—H11B	109.5
C2—C1—H1E	109.5	C12—C11—H11C	109.5
H1C—C1—H1E	109.5	H11A—C11—H11C	109.5
H1D—C1—H1E	109.5	H11B—C11—H11C	109.5
O1—C2—C1	107.6 (3)	O3—C12—C11	106.2 (3)
O1—C2—H2A	110.2	O3—C12—H12A	110.5
C1—C2—H2A	110.2	C11—C12—H12A	110.5
O1—C2—H2B	110.2	O3—C12—H12B	110.5
C1—C2—H2B	110.2	C11—C12—H12B	110.5
H2A—C2—H2B	108.5	H12A—C12—H12B	108.7
O2—C3—O1	120.3 (3)	O4—C13—O3	121.9 (3)
O2—C3—C4	126.2 (3)	O4—C13—C14	125.8 (3)
O1—C3—C4	113.5 (3)	O3—C13—C14	112.2 (3)
C5—C4—C9	116.4 (3)	C19—C14—C15	118.2 (3)
C5—C4—C3	123.1 (3)	C19—C14—C13	123.8 (3)
C9—C4—C3	120.5 (3)	C15—C14—C13	118.0 (3)
C6—C5—C4	121.0 (3)	C16—C15—C14	122.3 (3)
C6—C5—H5A	119.5	C16—C15—H15A	118.8
C4—C5—H5A	119.5	C14—C15—H15A	118.8
C5—C6—C7	121.8 (3)	C15—C16—C17	118.7 (3)
C5—C6—H6A	119.1	C15—C16—C20	120.9 (3)
C7—C6—H6A	119.1	C17—C16—C20	120.4 (3)
N1—C7—C6	121.1 (3)	N2—C17—C16	121.3 (3)
N1—C7—C8	119.7 (3)	N2—C17—C18	119.7 (3)
C6—C7—C8	119.1 (3)	C16—C17—C18	119.0 (3)
C9—C8—C7	117.5 (3)	C19—C18—C17	120.8 (3)
C9—C8—C10	121.9 (3)	C19—C18—H18A	119.6
C7—C8—C10	120.6 (3)	C17—C18—H18A	119.6
C8—C9—C4	124.2 (3)	C14—C19—C18	121.0 (3)
C8—C9—H9A	117.9	C14—C19—H19A	119.5
C4—C9—H9A	117.9	C18—C19—H19A	119.5
C8—C10—H10A	109.5	C16—C20—H20A	109.5
C8—C10—H10B	109.5	C16—C20—H20B	109.5
H10A—C10—H10B	109.5	H20A—C20—H20B	109.5
C8—C10—H10C	109.5	C16—C20—H20C	109.5
H10A—C10—H10C	109.5	H20A—C20—H20C	109.5
H10B—C10—H10C	109.5	H20B—C20—H20C	109.5
			
C3—O1—C2—C1	178.1 (3)	C13—O3—C12—C11	−177.7 (3)
C2—O1—C3—O2	1.4 (5)	C12—O3—C13—O4	−2.5 (5)
C2—O1—C3—C4	179.7 (3)	C12—O3—C13—C14	−179.4 (3)
O2—C3—C4—C5	−176.4 (3)	O4—C13—C14—C19	176.4 (4)
O1—C3—C4—C5	5.4 (5)	O3—C13—C14—C19	−6.9 (5)
O2—C3—C4—C9	2.0 (5)	O4—C13—C14—C15	−0.8 (6)
O1—C3—C4—C9	−176.2 (3)	O3—C13—C14—C15	176.0 (3)
C9—C4—C5—C6	1.4 (5)	C19—C14—C15—C16	0.8 (5)
C3—C4—C5—C6	179.9 (3)	C13—C14—C15—C16	178.1 (3)
C4—C5—C6—C7	−1.9 (6)	C14—C15—C16—C17	−1.4 (5)
C5—C6—C7—N1	179.2 (4)	C14—C15—C16—C20	−179.6 (3)
C5—C6—C7—C8	2.7 (6)	C15—C16—C17—N2	179.5 (4)
N1—C7—C8—C9	−179.6 (3)	C20—C16—C17—N2	−2.3 (6)
C6—C7—C8—C9	−3.0 (5)	C15—C16—C17—C18	1.3 (5)
N1—C7—C8—C10	2.9 (5)	C20—C16—C17—C18	179.5 (4)
C6—C7—C8—C10	179.4 (3)	N2—C17—C18—C19	−178.9 (4)
C7—C8—C9—C4	2.8 (5)	C16—C17—C18—C19	−0.7 (6)
C10—C8—C9—C4	−179.7 (3)	C15—C14—C19—C18	−0.1 (6)
C5—C4—C9—C8	−2.0 (5)	C13—C14—C19—C18	−177.2 (4)
C3—C4—C9—C8	179.6 (3)	C17—C18—C19—C14	0.0 (6)

### Hydrogen-bond geometry (Å, °)

**Table:**

*D*—H···*A*	*D*—H	H···*A*	*D*···*A*	*D*—H···*A*
N1—H1A···O2^i^	0.86	2.33	3.023 (5)	138
N1—H1B···N2	0.86	2.61	3.242 (5)	131
N2—H2C···O2^i^	0.86	2.35	3.160 (4)	157
N2—H2D···O4^ii^	0.86	2.15	2.967 (4)	158

Symmetry codes: (i) *x*−1, *y*−1, *z*; (ii) *x*, *y*−1, *z*.
